# Explainable artificial intelligence prediction-based model in laparoscopic liver surgery for segments 7 and 8: an international multicenter study

**DOI:** 10.1007/s00464-024-10681-6

**Published:** 2024-02-05

**Authors:** Victor Lopez-Lopez, Zeniche Morise, Mariano Albaladejo-González, Concepción Gomez Gavara, Brian K. P. Goh, Ye Xin Koh, Sijberden Jasper Paul, Mohammed Abu Hilal, Kohei Mishima, Jaime Arthur Pirola Krürger, Paulo Herman, Alvaro Cerezuela, Roberto Brusadin, Takashi Kaizu, Juan Lujan, Fernando Rotellar, Kazuteru Monden, Mar Dalmau, Naoto Gotohda, Masashi Kudo, Akishige Kanazawa, Yutaro Kato, Hiroyuki Nitta, Satoshi Amano, Raffaele Dalla Valle, Mario Giuffrida, Masaki Ueno, Yuichiro Otsuka, Daisuke Asano, Minoru Tanabe, Osamu Itano, Takuya Minagawa, Dilmurodjon Eshmuminov, Irene Herrero, Pablo Ramírez, José A. Ruipérez-Valiente, Ricardo Robles-Campos, Go Wakabayashi

**Affiliations:** 1 Department of General, Visceral and Transplantation Surgery, Clinic and University Hospital Virgen de La Arrixaca, IMIB-ARRIXACA, El Palmar, Murcia, Spain; 2 Department of Surgery, Fujita Health University School of Medicine Okazaki Medical Center, Okazaki, Aichi Japan; 3 Department of Information and Communication Engineering, Murcia University, Murcia, Spain; 4 Department of HPB Surgery and Transplants, Vall d’Hebron University Hospital, Barcelona Autonomic University, Barcelona, Spain; 5 Department of Hepatopancreatobiliary and Transplant Surgery, Singapore General Hospital and National Cancer Centre Singapore, Singapore, Singapore; 6 Surgery Academic Clinical Programme, Duke-National University of Singapore Medical School, Singapore, Singapore; 7 Department of Surgery, Fondazione Poliambulanza Istituto Ospedaliero, Brescia, Italy; 8 Department of Surgery, University Hospital Southampton NHS Foundation Trust, Southampton, UK; 9 Department of Surgery, Ageo Central General Hospital, Ageo, Japan; 10 Serviço de Cirurgia do Fígado, Divisão de Cirurgia do Aparelho Digestivo, Departamento de Gastroenterologia, Faculdade de Medicina, Hospital das Clínicas HCFMUSP, Universidade de São Paulo, São Paulo, Brazil; 11 Department of General, Pediatric and Hepatobiliary-Pancreatic Surgery, Kitasato University School of Medicine, Sagamihara, Japan; 12 Department of General Surgery, School of Medicine, Clínica Universidad de Navarra, University of Navarra, Pamplona, Spain; 13 Department of Surgery, Fukuyama City Hospital, Hiroshima, Japan; 14 Department of Surgery, National Cancer Center Hospital East, Kashiwa, Japan; 15 Department of Hepato-Biliary-Pancreatic Surgery, Osaka City General Hospital, Osaka, Japan; 16 Department of Surgery, Fujita Health University, Toyoake, Japan; 17 Department of Surgery, Iwate Medical University, Iwate, Japan; 18 General Surgery Unit, Parma University Hospital, Parma, Italy; 19 Second Department of Surgery, Wakayama Medical University, 811-1 Kimiidera, Wakayama City, Wakayama Japan; 20 Department of Surgery, Toho University, Tokyo, Japan; 21 Department of Hepatobiliary and Pancreatic Surgery, Graduate School of Medicine, Tokyo Medical and Dental University, Tokyo, Japan; 22 Department of Hepato-Biliary-Pancreatic and Gastrointestinal Surgery, School of Medicine, International University of Health and Welfare, Chiba, Japan; 23 Department of Surgery and Transplantation, University Hospital Zurich and University of Zurich, Zurich, Switzerland; 24 Department of Surgery, Getafe University Hospital, Madrid, Spain

**Keywords:** Artificial intelligence, Making decision, Minimally invasive surgery, Liver resection

## Abstract

**Background:**

Artificial intelligence (AI) is becoming more useful as a decision-making and outcomes predictor tool. We have developed AI models to predict surgical complexity and the postoperative course in laparoscopic liver surgery for segments 7 and 8.

**Methods:**

We included patients with lesions located in segments 7 and 8 operated by minimally invasive liver surgery from an international multi-institutional database. We have employed AI models to predict surgical complexity and postoperative outcomes. Furthermore, we have applied SHapley Additive exPlanations (SHAP) to make the AI models interpretable. Finally, we analyzed the surgeries not converted to open versus those converted to open.

**Results:**

Overall, 585 patients and 22 variables were included. Multi-layer Perceptron (MLP) showed the highest performance for predicting surgery complexity and Random Forest (RF) for predicting postoperative outcomes. SHAP detected that MLP and RF gave the highest relevance to the variables “resection type” and “largest tumor size” for predicting surgery complexity and postoperative outcomes. In addition, we explored between surgeries converted to open and non-converted, finding statistically significant differences in the variables “tumor location,” “blood loss,” “complications,” and “operation time.”

**Conclusion:**

We have observed how the application of SHAP allows us to understand the predictions of AI models in surgical complexity and the postoperative outcomes of laparoscopic liver surgery in segments 7 and 8.

**Supplementary Information:**

The online version contains supplementary material available at 10.1007/s00464-024-10681-6.

Artificial Intelligence (AI) has great potential to aid decision making in medicine [1]. However, the lack of transparency and interpretability of many AI models is a huge concern for their use in real medical applications, especially in black-box models [2]. One of the great challenges we currently face is to define the AI models that best represent our medical needs. Not only does it consist of data analysis, but the objective is also to integrate this type of methodology so that it has a logic in our daily surgical practice and allows us to make better decisions, especially in our more complex surgeries [3–6].

An example of a surgical technique with a high level of difficulty is represented by minimally invasive liver surgery of the posterosuperior segments [7–9]. This procedure has been one of the latest to be developed due to its complexity and the need for extensive experience and training in laparoscopic liver surgery. Although, in the last decade, different centers have gained a vast amount of experience with this procedure, it continues to be very challenging. Furthermore, not all lesions and patients are the same; therefore, the complexity depends not only on whether the tumor is in a posterosuperior segment, but also on many other factors related to the patient’s characteristics, like the size of the lesion, the location, the proximity to the hepatic veins, or the type of resection, which will influence both the complexity of the surgery and the postoperative outcomes.

To solve this issue, we have developed AI models to assist decision making in the more complex segments of laparoscopic liver surgery, and we have employed explainable AI through Shapley Additive exPlanations (SHAP) to understand the reasoning followed by the model [10]. SHAP is a game theoretic approach to explain the output of any AI model. It combines optimal credit allocation and local explanations employing the classic Shapley values from game theory. SHAP allows us to calculate how much our predictor variables contribute to the final prediction and the overall importance of the variables to the AI model.

## Methods

### Study design

From an international multi-institutional database from 19 hospitals with experience in minimally invasive liver surgery, we collected 585 patients with lesions (metastases and malignant or benign primary tumors) located in segments 7 and 8 operated by minimally invasive liver surgery. On this cohort of patients, we tested different AI algorithms to assist in decision-making. The main objective of the study was to analyze whether the final AI model developed would be useful for predicting the complexity of the procedure to be performed and the postoperative outcomes to be obtained depending on the characteristics of each patient and the lesion. The secondary objective was to predict which surgeries would be converted to open surgery. The institutional review board at the Clinic and University Virgen de la Arrixaca Hospital (Murcia, Spain) approved this study (Internal Protocol Code: 2021-4-8-HCUVA).

The variables included in the analysis were age, sex, American Society of Anesthesiologist (ASA), body mass index (BMI), Charlson comorbidity index, liver disease, tumor location, type of resection, minimally invasive approach (hand-assisted/hybrid or pure), previous hepatectomy, neoadjuvant chemotherapy, proximity to major vessel, largest tumor size, number of lesions, Child Pugh B, blood loss, surgical time, Pringle time, postoperative complications, readmission, hospital stay, and conversion to open. Three of these initial variables were related to the complexity of the surgery and another three to the postoperative outcomes. We combined the three complexity variables to create an additional variable that summarized the complexity of a surgery. We applied the same approach to the postoperative outcome variables. This process is described in more detail in the following subsections. We calculated the Pearson Correlation Coefficient to describe the correlation of the complexity and outcome variables with their new aggregation variables. We employed this correlation to illustrate the aggregated variables’ dependence on the original variables. After that, we used the dataset without the complexity and outcome variables, and we predicted our summary variable of complexity and outcome through AI models (including a grid search to configure the models). Finally, we analyzed the surgeries not converted to open versus those converted to open.

### Development of the AI models

The prediction of the complexity and surgery outcome was performed using AI models. In both problems, we only used surgeries that were not converted to open as they were analyzed separately afterwards. The non-converted surgeries were split into training (70%) and test (30%) datasets. The training set was used as input for the database transformations, the hyperparameter tuning, the model selection, training of the final model, and applying SHAP. The test set received the same transformations as the training set, though the training set was never used as reference data in any of the transformations performed on the test set. Therefore, the test set was only used for the final test evaluation to avoid any biases in our evaluation that could jeopardize the results. The first database transformation consisted of applying One Hot Encoding to the qualitative variables of the training and test sets. The One Hot Encoding was essential because the AI models cannot utilize categorical data. Subsequently, we performed the imputation of the missing values using k-Nearest Neighbors (KNN) imputation. We trained this imputer with the training set, and it imputed the missing values of both sets, replacing each missing value with the value of the most similar surgery in the training set.

### Definitions of complexity and postoperative outcomes

The next step was to create variables that summarized the complexity and outcomes of a surgery. We created a new variable, namely “complexity” that summarizes the complexity of the operation based on “blood loss,” “surgical time,” and “Pringle time” by applying a z-score standardization. The goal of standardization was to guarantee that each of the three variables was equally considered during the creation of the new variable. Without this procedure, the importance of one or two variables in creating the new variable could be considerably reduced due to the other variable having larger values. Then, we added the three variables and normalized the resulting variable to the range [0, 1] (the minimum value became 0, and the maximum value became 1). This transformation was done using the training sets as the reference data and applied to the training and test sets. Therefore, the mean, standard deviation, minimum, and maximum required were obtained only from the training set. The created variable “complexity” has a Pearson correlation of 0.67 with “blood loss,” 0.54 with “surgical time,” and 0.73 with “Pringle time.”

We applied this same process with the variables “complications,” “readmission,” and “hospital stay” to create the variable that summarizes the “postoperative outcomes” of laparoscopic liver surgery. The last preprocessing step was standardizing the predictor variables (the preoperative variables). This standardization is particularly recommendable for AI models that employ weights or distances. We considered this standardization as an optional step; thus, we sought the best configuration of the AI models in the hyperparameter tunning with and without applying the standardization. Finally, the “outcome” variable had a Pearson correlation of 0.76 with “complications,” 0.57 with “readmission,” and 0.65 with “hospital stay.”

### Configuration and evaluation of the AI models

To select the best configuration of each model and the best model, we employed a grid search with a tenfold cross-validation using the training set. In a tenfold cross-validation, the available dataset is partitioned into ten disjoint subsets of equal size (folds), performing a random sampling without replacement. The model is trained using nine subsets representing the training and evaluated in the remaining fold, denoted as the validation set. This procedure is repeated until each of the ten subsets has served as a validation set for each *model* and configuration. In the configuration and selection of the best model, we evaluated KNN, Multi-layer Perceptron (MLP), Random Forest (RF), Adaboost, Linear Discriminant Analysis (LDA), Support Vector Regression (SVR), ElasticNet, and Linear Regressor. All the implementations utilized for these models were from the Scikit-learn library of Python. The error metrics reported for each of these models were Mean Absolute Error (MAE), Mean-Square Error (MSE), and Root-Mean-Square Error (RMSE). We employed these metrics because they are well recognized in the state of the art for regression problems. Among these metrics, we optimize the MAE metric.

**Fig. 1:**
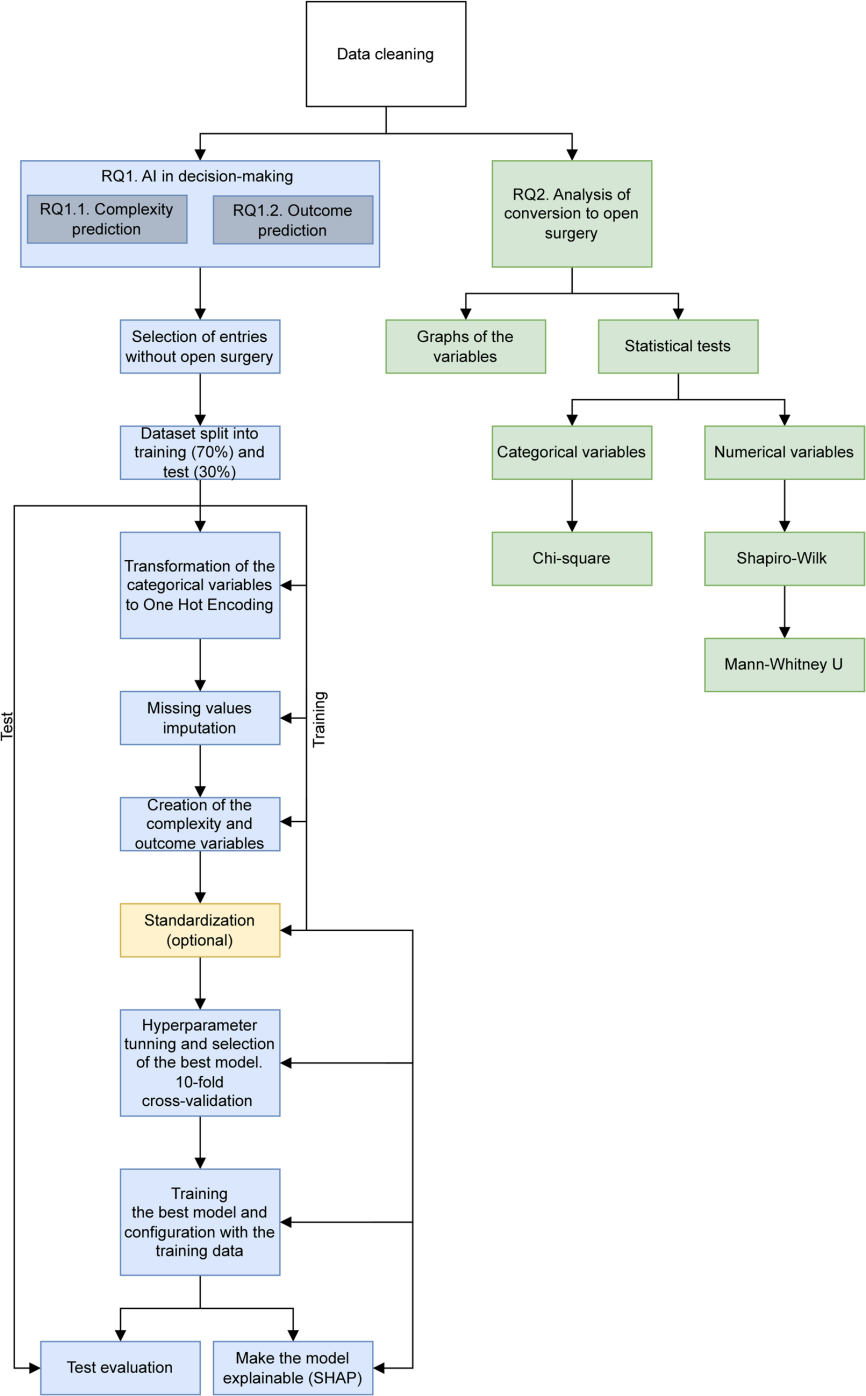
Methodology followed to answer the research questions

After the model configuration and selection, the best AI model (the model and configuration that reported the highest MAE in the cross-validation) was trained with all the training set and evaluated in the test set, which was not used until this moment. Finally, to make the model interpretable, we employed SHAP, obtaining the average general importance given by the AI model to each variable. This information offers us a general understanding of the AI model and how it considers the input variables to predict the surgery’s complexity and outcome. Furthermore, using SHAP, we also obtained the contribution of each variable in a specific instance prediction. In that case, SHAP explains why the model makes the prediction showing how the values of the input variables influence the prediction. In a real application of the developed models, the contributions of the variables provide the reasoning followed by the AI model when predicting the complexity or the outcomes of surgery (Fig. 1).

**Fig. 2:**
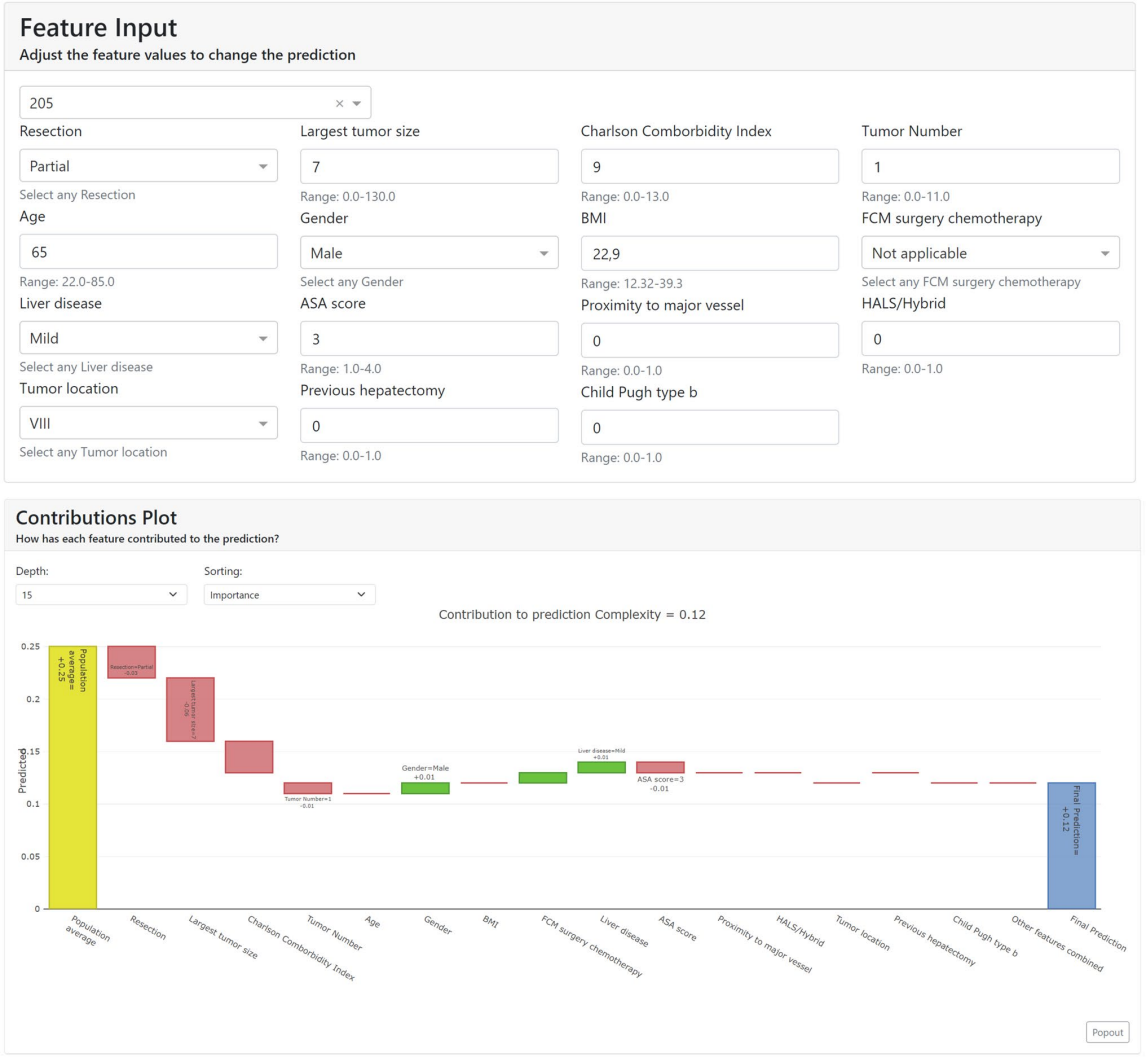
Prediction risk calculator created using the Shapash library the Python programming language. This library generates an interactive dashboard that allows the prediction of each new case

Finally, a prediction view has been created using the Shapash library (https://shapash.readthedocs.io/en/latest/) of the Python programming language. This library generates an interactive dashboard that allows the prediction of each new case. This dashboard offers interactively and through a web interface to observe the importance of the variables, to visualize the operations of the training set and to enter the data of new operations, receiving in this last case the prediction of the model and the contribution of each variable to the prediction (Fig. 2).

### Conversion to open analysis

In addition, we analyzed the surgeries not converted to open versus those converted to open. This analysis aims to find the reasons for conversion to open surgeries. To do this, we analyzed the distribution of each variable in function of the conversion to open surgery through graphs. Furthermore, we applied statistical tests to find significant differences in each variable depending on the conversion to open surgery. For the categorical variables, we employed the Chi-square test. For the numerical variables, we first evaluated if the samples fit a normal distribution through the Shapiro–Wilk test. This test reported that at least one distribution in each variable does not fit a normal distribution, and therefore, we applied the non-parametric test of Mann–Whitney *U*.

## Results

### Predicting the complexity of the surgery

**Fig. 3:**
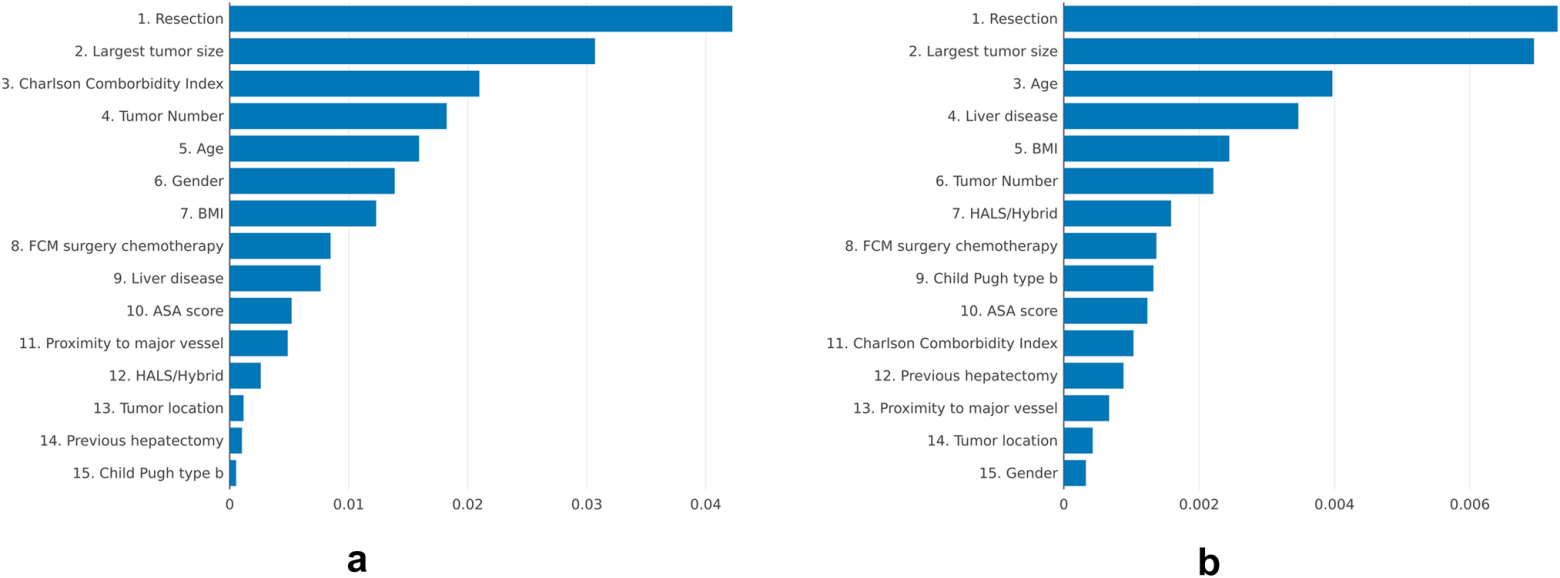
**a** Importance of each variable for the MLP to predict the complexity of the surgery; **b** Importance of each variable for Random Forest to predict the outcomes of the surgery

In the prediction of the complexity of the surgery, the performance of the best configuration of each model in the hyperparameter tuning is shown in Supplementary Table 1. The MLP reported the best performance without scaling the database. This result is surprising because MLP usually improves its performance with the scaled data because neural networks employ different weights that adjust during training. Besides, we observed that black-box models (MLP, RF, and Adaboost) obtained higher performance than simpler models. Figure 3a shows the importance of the variables given by the model to each variable in the overall training set. This figure gives the surgeons a general understanding of the AI model behavior. We observe that the type of resection is the most important variable for the AI model to predict complexity with a SHAP value of 0.042. “Largest tumor size” and “Charlson Combordity Index” are also relevant to the model with SHAP values 0.031 and 0.021, respectively. The rest of the variables have an importance lower than half of the most important variable.

**Fig. 4:**
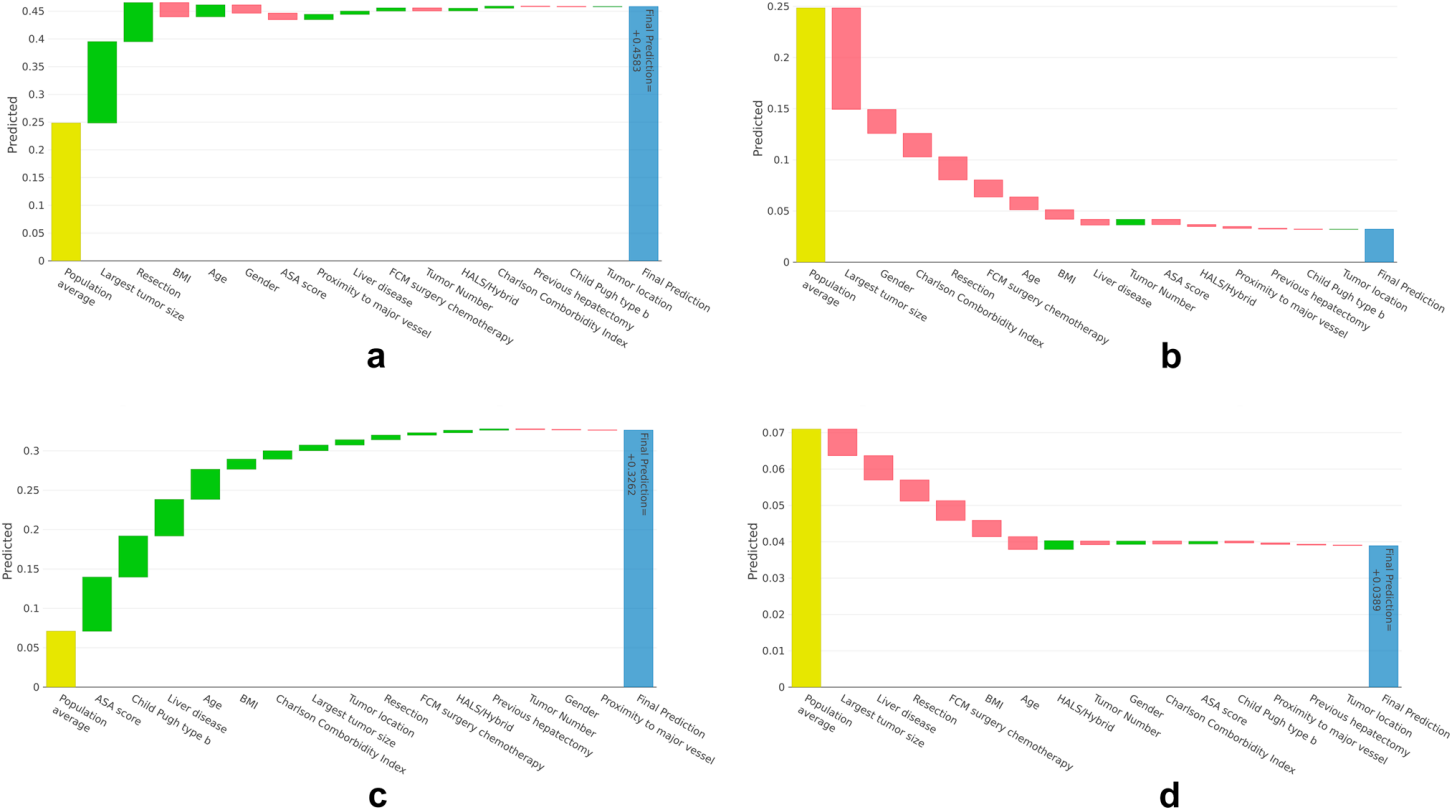
Representation of 4 examples according to the values of each variable entered in the calculator. **a** Prediction of the complexity of the surgery: “ASA score”: 3, “age”: 76, “BMI”: 33, “Charlson Comborbidity Index”: 6, “Child Pugh type b”: “False,” “FCM surgery chemotherapy”: “Yes,” “gender”: “female,” “HALS/Hybrid”: “False,” “Largest tumor size”: 100, “Liver disease”: “Mild”, “Previous hepatectomy”: “False,” “Proximity to major vessel”: “True”, “Resection”: “Sectionectomy and more,” “Tumor number”: 1, and “Tumor location”: “VII”; **b** Prediction of the complexity of the surgery: “ASA score”: 3, “age”: 61, “BMI”: 29.7, “Charlson Comborbidity Index”: 8, “Child Pugh type b”: “False,” “FCM surgery chemotherapy”: “True,” “gender”: “female”; **c** Prediction of the postoperative outcomes of the surgery: “ASA score”: 4, “age”: 48, “BMI”: 23.53, “Charlson Comborbidity Index”: 5, “Child Pugh type b”: “True,” “gender”: “male,” “HALS/Hybrid”: “False,” “Largest tumor size”: 30, “Liver disease”: “Moderate to severe,” “Previous hepatectomy”: “False,” “Proximity to major vessel”: “False,” “Resection”: “Partial,” “Tumor number”: 1, and “Tumor location”: “VIII”; **d** Prediction of the postoperative outcomes of the surgery: “ASA score”: 1, “age”: 46, “BMI”: 29.33, “Charlson Comborbidity Index”: 6, “Child Pugh type b”: “False,” “FCM surgery chemotherapy”: “True,” “gender”: “female,” “HALS/Hybrid”: “true,” “Largest tumor size”: 20, “Liver disease”: “None,” “Previous hepatectomy”: “False,” “Proximity to major vessel”: “False,” “Resection”: “Partial,” “Tumor number”: 1, and “Tumor location”: “VII.”

Figure 4a and b illustrates two cases, demonstrating how specific patient and lesion data influence the prediction of surgical complexity through our model and SHAP in the dashboard (Fig. 2). Therefore, both figures show the contribution of each of these variables for the final prediction of the degree of complexity that these individualized cases will have. In both figures, the “population average” column indicates the default value predicted by the model without considering any variable. After this column, the figure shows how each variable increases (green) or decreases (red) the final prediction (last column). Both figures give the surgeons the reasoning followed by the model for predicting two surgeries. In Fig. 4a, we appreciate that the main reason the model predicted a complexity of 0.4583 is that the “largest tumor size” is 100 mm and the “resection” is “sectionectomy and more,” which increases the prediction. In contrast, Fig. 4b shows that the "largest tumor size" of 2 mm decreases the final prediction. Most of the other variables also contribute to decreasing the complexity.

### Predicting the outcome of the surgery

The performance of the best configuration of each model in the hyperparameter tuning for predicting the outcome of the surgery is shown in Supplementary Table 2. For this problem, the best performance was obtained by RF without scaling the data. In this case, we selected RF as the best model, unlike in the complexity prediction; here, the best model does not have the best performance on all error metrics. We chose RF because it achieved the best MAE, which is the optimization metric we selected in the methodology. Again, the selected model is a black-box model, and we appreciate that the most explainable and interpretable models achieved lower performances than most complex models. Figure 3b shows the importance of the variables given by RF to each variable in the overall training set. We observed that for the prediction of the outcome of the surgery, the “resection” and “largest tumor size” again are the two most important variables with SHAP values 0.0073 and 0.0069, respectively. The age has a SHAP value of 0.004 and the rest of the variables have an importance lower than half of the most important variable.

Figure 4c and d exemplifies two cases in which, according to the specific data of each patient and lesion that we introduce in our model and SHAP through the dashboard (Fig. 2), we show the contribution of each of these variables for the final prediction of the postoperative outcomes that we are going to have in each individualized case. Figure 4c represents how most of the variables increase the final prediction in contrast to Fig. 4d, where most of the variables decrease the final prediction. In these two examples, there is no single variable that stands out for its contribution to the final prediction, but rather many variables contribute to the prediction.

### Interactive dashboard

Finally, as part of our implementation, we introduced MLP and RF models into an interactive dashboard. This integrated dashboard shows the power of SHAP, allowing the surgeons to introduce the data of one patient and receive predictions for both surgical complexity and postoperative outcomes. Furthermore, the dashboard provides visual representations of each feature’s importance and influence (contribution) in a particular surgery, as Fig. 2 shows. Through the contribution of each variable, the surgeon can understand the reasoning followed by the AI models for a particular complexity and postoperative outcome prediction. Therefore, the surgeon can make a more informed decision for each surgery, knowing the complexity, the postoperative outcome, and how patient variables influence in the surgery.

### Conversion to open analysis

**Fig. 5:**
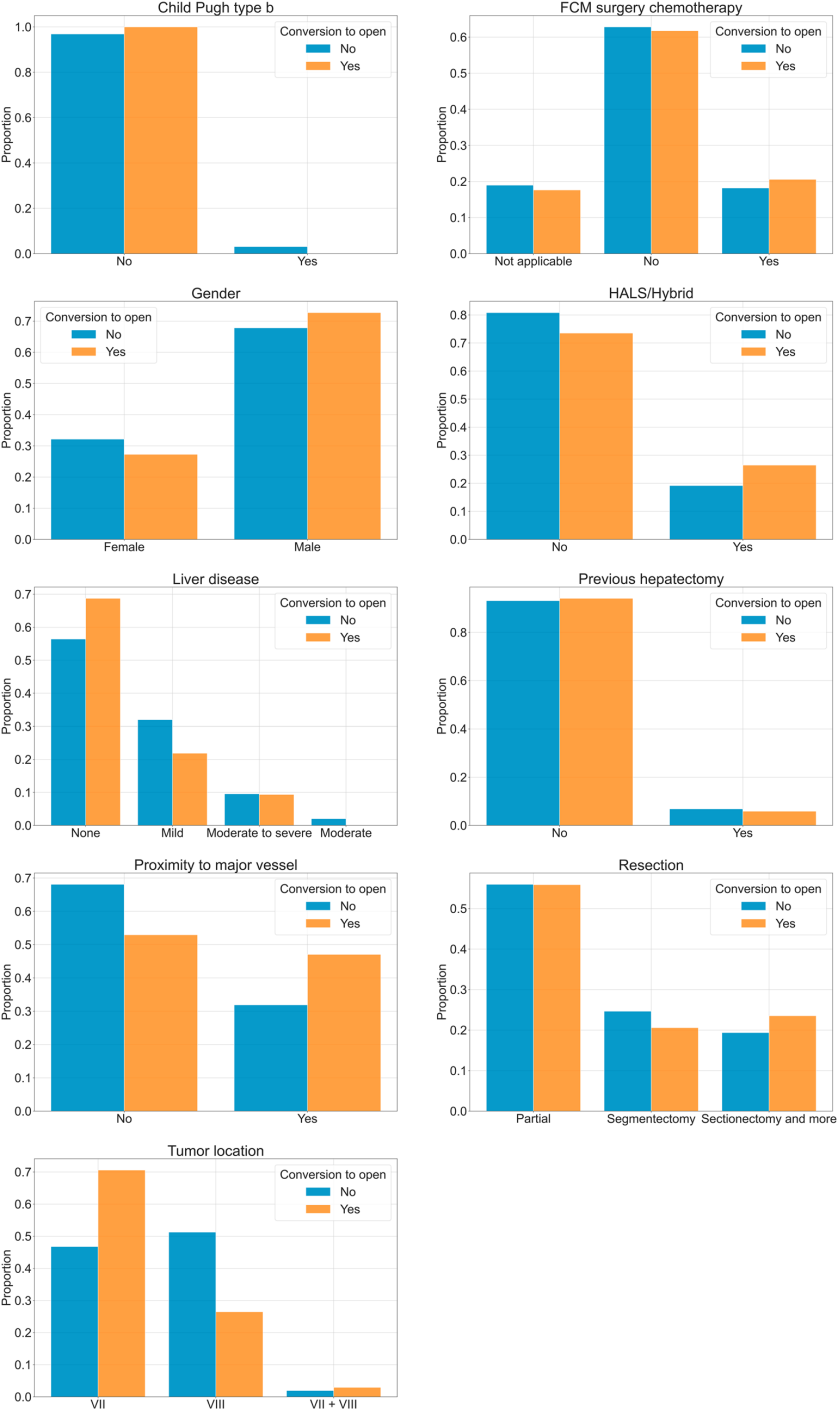
Distribution of each categorical variable by conversion to open surgery

In order to find differences in the numerical variables between surgeries converted and non-converted to open, we developed the graphs included in Fig. 5. These graphs show the distribution of each variable separated by the conversion and non-conversion to open surgery. We detected differences in the distribution of the variable “tumor location” depending on the conversion to open surgery. Furthermore, Supplementary Table 3 contains the results of applying the Chi-square test to the qualitative variables. This table indicates that the Chi-square test found significant differences with a *P* value of 0.0434 in the variable “tumor location” as a function of the conversion to open surgery.

On the other hand, the distribution of each numerical variable as a function of conversion to open surgery is shown through the box and violin plots included in Supplementary Fig. 1. We can appreciate differences in the “ASA score,” “blood loss,” “complications,” and “operation time” variables. Table 1 shows the mean of each variable depending on conversion to open surgery and the results of the Mann–Whitney *U* statistical test. In this table, we appreciate that the differences detected in “blood loss,” “complications,” and “operation time” through the graphs are statistically significant. In contrast, the mean differences of the variable “ASA score” do not show remarkable changes depending on the conversion to open surgery, and also, the Mann–Whitney *U* did not find significant differences.

**Table 1:** Summary of the means and Mann–Whitney *U* test for numerical variables depending on the conversion to open surgery

Variable	Mean			Mann–Whitney *U* test	
	Converted to open	Non-converted to open	Difference	Value	*P* value
Complications	0.8158	0.3388	0.477	13497.5	0.0001
Blood loss	1297.886	303.3081	994.5776	12850.5	0.0001
Operation time	362.6486	304.7749	57.8737	12466	0.0132
Hospital stay	15.5	9.7523	5.7477	11579.5	0.0688
ASA score	2.0588	2.2231	− 0.1643	7396	0.0807
Largest tumor size	37.0294	30.5457	6.4837	10185	0.0877
BMI	26.0999	24.7073	1.3926	9494.5	0.1128
Age	63.5	65.501	− 2.001	7811.5	0.3248
Charlson Comborbidity Index	6.8125	6.3157	0.4968	8551.5	0.3972
Tumor number	1.1765	1.4303	− 0.2538	8260	0.5314
Pringle time	47.9211	49.5018	− 1.5808	10283.5	0.9886

## Discussion

This is the first AI-based model that categorizes surgical procedure complexity and predicts surgical outcomes in patients undergoing laparoscopic liver resection for lesions located in segments 7 and 8. We have observed how the application of SHAP allows us to understand the overall behavior of an AI model in predicting the complexity and the postoperative outcomes of laparoscopic liver surgery. SHAP allows surgeons to learn which factors are important in predicting outcomes that predict operative results in the AI model. In addition, the contribution of each variable to the final prediction allows surgeons to understand the reasoning followed in predicting a particular surgery. This transparency of the AI models developed allows their application to assist the decision-making process in complex laparoscopic liver surgery.

AI has been incorporated into our day-to-day lives influencing many of the decisions we make, in many cases without us being aware of it ourselves [11, 12]. It is important to differentiate between AI, which is primarily based on data analysis, and machine learning, which focuses on models with learning capabilities [13].

The potential of machine learning and AI is already used in surgery, with the aim of exploring how it can improve our decision making in the future when faced with a given clinical situation [14–16]. In hepatobiliary surgery, different groups have successfully developed models to improve perioperative management of patients with hepatocellular carcinoma and predict both complications and recurrence patterns [17]. For example, to predict the risk of posthepatectomy liver failure, several artificial neural network models to help surgeons identify those patients at intermediate and high risk have been described [18]. Our group defined a risk-scoring model useful to estimate the patients’ level of risk based on the initial presentation and bile duct injury type and detailed how the patient’s risk category may be used to determine the appropriate management [19].

Research on the usefulness of AI focused on laparoscopic liver surgery is anecdotal [20]. The most important experience was described by Ruzzenente et al., where they used a RF model among the four most important difficulty scoring systems to rank technical complexity in laparoscopic liver resections and predict five individual interoperative and postoperative outcomes, assuming that the more difficult a resection is the higher the incidence of worse outcomes [21].

Many factors have been related to the complexity and postoperative outcomes of laparoscopic liver surgery without reaching a clear consensus [22–24]. Of all of them, most authors highlight the importance of the size and number of lesions, body mass index, characteristics of the liver, etiology of the surgical indication, relationship with the pedicle or the hepatic veins, the type of resection, neoadjuvant chemotherapy, or history of a previous hepatectomy. So, although they are all obviously important, we still do not know which are really the most determining factors and the specific weight of each one of them. Furthermore, it is not the same to perform a minimally invasive approach in a favorable segment of the liver compared to a more unfavorable one. In this sense, some authors have considered segments 6,7,8, 4a, and even 1 as posterosuperior segments, when the approach and the results are not comparable [25–28]. In fact, segments 4a,6 and 1 are technically less complex. For this reason, our group focused exclusively on segments 7 and 8 because they share the similarities in technical difficulties.

In this prediction model, the variables that on average have the greatest importance are those that a surgeon could give importance to preoperatively. On the one hand, Fig. 3a and b reflects the importance of the variables in general for all the laparoscopic resections in segments 7 and 8 that we have analyzed. Significance has a positive value, so if a partial resection decreases the complexity of the operation, and a segmentectomy increases it, the model will return a positive significance. In short, they represent the relevance of the variables, regardless of whether the effect of the possible values of the variable is positive or negative in the prediction. These figures would indicate what a surgeon would predict for each variable. In general, they are related to the complexity of the surgery and to obtaining the best possible postoperative results, respectively.

On the other hand, in Figs. 2 and 4, our model adds the characteristics of each case and provides us an individualized prediction based on the first global analysis that we have performed. For a particular surgery, each variable takes one of the possible values and has a specific effect (contribution) on the prediction. Therefore, the model is explaining us why that particular decision has been made and how the value of each variable has influenced the prediction of the complexity or outcomes of the surgery.

Another key point is related to the probability of conversion to open surgery. There are many factors that influence the risk of the conversion during a laparoscopic liver resection [29]. The posterosuperior segments have been associated with a higher conversion rate than other segments. In this sense, according to our model, those surgeries in segment 7 have a statistically significant higher probability of conversion, especially when associated with greater blood loss and surgical time. This shows that, in minimally invasive liver surgery, resections in segment 7 are more likely to end up in a conversion to open surgery and how the location of the lesions in this segment is more determinant than any other factor.

## Conclusion

This methodology has demonstrated its potential usefulness in a very specific homogeneous cohort of patients characterized by its difficulty. There are new challenges to be faced in order to validate this model and better understand the extent of its feasibility. It is necessary to perform this type of analysis on much larger patient samples that homogeneously represent a surgical technique. Our goal should be to try to predict, depending on the exact location of the tumor, the patient’s characteristics, and the surgical technique required, the chances of success adjusted to the case. According with the AI complexity prediction, we can decide which type of surgeon is going to perform the surgery based on their learning curve, and we can inform the patients that depending on their characteristics, the degree of difficulty, and the percentage of complications will be higher.

This dashboard using SHAP is an example of AI interpretability potential in the field of highly complex surgery, where AI can play a key role in aiding decision making. However, many researchers continue to bypass this last step of including explainable AI techniques, making these models difficult to use and to trust their predictions. It is true that there is still a need for further exploration of this technology, which must always be accompanied by the experience of surgeons for its predictions to make sense. Even so, there is no doubt that we must adapt to this new methodology, as in the next years, it will be part of our daily lives.

### Supplementary Information

Below is the link to the electronic supplementary material.

Supplementary file1 (DOCX 586 KB)

## Abbreviations

AI: Artificial intelligence
KNN: K-nearest neighbors
LDA: Linear discriminant analysis
MAE: Median absolute error
RF: Random forest
SHAP: SHapley Additive exPlanations
SVR: Support vector regression
MLP: Multi-layer perceptron
MSE: Mean-square error
RMSE: Root-mean-square error
