# Correction: Explainable artificial intelligence prediction-based model in laparoscopic liver surgery for segments 7 and 8: an international multicenter study

**DOI:** 10.1007/s00464-024-10787-x

**Published:** 2024-03-19

**Authors:** Victor Lopez-Lopez, Zeniche Morise, Mariano Albaladejo-González, Concepción Gomez Gavara, Brian K. P. Goh, Ye Xin Koh, Sijberden Jasper Paul, Mohammed Abu Hilal, Kohei Mishima, Jaime Arthur Pirola Krürger, Paulo Herman, Alvaro Cerezuela, Roberto Brusadin, Takashi Kaizu, Juan Lujan, Fernando Rotellar, Kazuteru Monden, Mar Dalmau, Naoto Gotohda, Masashi Kudo, Akishige Kanazawa, Yutaro Kato, Hiroyuki Nitta, Satoshi Amano, Raffaele Dalla Valle, Mario Giuffrida, Masaki Ueno, Yuichiro Otsuka, Daisuke Asano, Minoru Tanabe, Osamu Itano, Takuya Minagawa, Dilmurodjon Eshmuminov, Irene Herrero, Pablo Ramírez, José A. Ruipérez-Valiente, Ricardo Robles-Campos, Go Wakabayashi

**Affiliations:** 1 Department of General, Visceral and Transplantation Surgery, Clinic and University Hospital Virgen de La Arrixaca, IMIB-ARRIXACA, El Palmar, Murcia, Spain; 2 Department of Surgery, Fujita Health University School of Medicine Okazaki Medical Center, Okazaki, Aichi Japan; 3 Department of Information and Communication Engineering, Murcia University, Murcia, Spain; 4 Department of HPB Surgery and Transplants, Vall d’Hebron University Hospital, Barcelona Autonomic University, Barcelona, Spain; 5 Department of Hepatopancreatobiliary and Transplant Surgery, Singapore General Hospital and National Cancer Centre Singapore, Singapore, Singapore; 6 Surgery Academic Clinical Programme, Duke-National University of Singapore Medical School, Singapore, Singapore; 7 Department of Surgery, Fondazione Poliambulanza Istituto Ospedaliero, Brescia, Italy; 8 Department of Surgery, University Hospital Southampton NHS Foundation Trust, Southampton, UK; 9 Department of Surgery, Ageo Central General Hospital, Ageo, Japan; 10 Serviço de Cirurgia do Fígado, Divisão de Cirurgia do Aparelho Digestivo, Departamento de Gastroenterologia, Faculdade de Medicina, Hospital das Clínicas HCFMUSP, Universidade de São Paulo, São Paulo, Brazil; 11 Department of General, Pediatric and Hepatobiliary-Pancreatic Surgery, Kitasato University School of Medicine, Sagamihara, Japan; 12 Department of General Surgery, School of Medicine, Clínica Universidad de Navarra, University of Navarra, Pamplona, Spain; 13 Department of Surgery, Fukuyama City Hospital, Hiroshima, Japan; 14 Department of Surgery, National Cancer Center Hospital East, Kashiwa, Japan; 15 Department of Hepato-Biliary-Pancreatic Surgery, Osaka City General Hospital, Osaka, Japan; 16 Department of Surgery, Fujita Health University, Toyoake, Japan; 17 Department of Surgery, Iwate Medical University, Iwate, Japan; 18 General Surgery Unit, Parma University Hospital, Parma, Italy; 19 Second Department of Surgery, Wakayama Medical University, 811-1 Kimiidera, Wakayama City, Wakayama Japan; 20 Department of Surgery, Toho University, Tokyo, Japan; 21 Department of Hepatobiliary and Pancreatic Surgery, Graduate School of Medicine, Tokyo Medical and Dental University, Tokyo, Japan; 22 Department of Hepato-Biliary-Pancreatic and Gastrointestinal Surgery, School of Medicine, International University of Health and Welfare, Chiba, Japan; 23 Department of Surgery and Transplantation, University Hospital Zurich and University of Zurich, Zurich, Switzerland; 24 Department of Surgery, Getafe University Hospital, Madrid, Spain

**Correction to: Surgical Endoscopy** 10.1007/s00464-024-10681-6

The original online version of this article was revised to correct the presentation of the name of coauthor Mariano Albaladejo-González.

The original article has been corrected.

