# Supplementary material for: Explainable artificial intelligence prediction-based model in laparoscopic liver surgery for segments 7 and 8: an international multicenter study
Source: Surg Endosc. 2024 Feb 5;38(5):2411–22. doi: 10.1007/s00464-024-10681-6 (PMC11078826; doi:10.1007/s00464-024-10681-6)

**Supplementary Information for Manuscript “Explainable Artificial Intelligence Prediction Based Model in Advanced Laparoscopic Liver Surgery: An International Multicenter Study”**

Victor Lopez-Lopez, Zeniche Morise Mariano Albadalejo-González, Concepción Gomez Gavara, Brian KP Goh, Ye Xin KO, Sijberden Jasper Paul, Mohammed Abu Hilal, Kohei Mishima, Jaime Arthur Pirola Krürger Paulo Herman, Alvaro Cerezuela, Roberto Brusadin, Takashi Kaizu, Juan Lujan, Fernando Rotellar, Kazuteru Monden, Mar Dalmau, Naoto Gotohda, Masashi Kudo, Akishige Kanazawa, Yutaro Kato, Hiroyuki Nitta, Satoshi Amano, Raffaele Dalla Valle, Mario Giuffrida, Masaki Ueno, Yuichiro Otsuka, Daisuke Asano, Minoru Tanabe, Osamu Itano, Takuya Minagawa, Dilmurodjon Eshmuminov, Irene Herrero, Pablo Ramírez, José A Ruipérez-Valiente, Ricardo Robles-Campos, and Go Wakabayashi

| Model | Database  scaled | RMSE | MSE | MAE |
| --- | --- | --- | --- | --- |
| MLP | False | 0.1394 | 0.0203 | 0.1045 |
| Random Forest | False | 0.1409 | 0.0205 | 0.1048 |
| Random Forest | True | 0.1409 | 0.0205 | 0.1049 |
| Adaboost | True | 0.1423 | 0.021 | 0.1071 |
| Adaboost | False | 0.1428 | 0.0211 | 0.1072 |
| KNN | True | 0.1472 | 0.0222 | 0.1073 |
| MLP | True | 0.1454 | 0.022 | 0.1075 |
| SVR | True | 0.1436 | 0.0211 | 0.109 |
| SVR | False | 0.1476 | 0.0225 | 0.1096 |
| ElasticNet | False | 0.1455 | 0.0218 | 0.1099 |
| Tree | True | 0.1493 | 0.023 | 0.1103 |
| Tree | False | 0.1493 | 0.023 | 0.1103 |
| ElasticNet | True | 0.1478 | 0.0225 | 0.1121 |
| Linear regressor | False | 0.1491 | 0.0229 | 0.1134 |
| Linear regressor | True | 0.1491 | 0.0229 | 0.1134 |
| KNN | False | 0.1538 | 0.0242 | 0.1135 |

**Supplementary table 1.** Performance obtained by the best configuration of each model in the 10-fold cross-validation to predict the complexity of the surgery. Once the MLP was trained with the entire training set without scaling, it obtained an RMSE of 0.1831, an MSE of 0.0335, and an MAE of 0.1132 in the test set. The performance achieved in the test set is good, allowing its application in real scenarios. However, the MLP model is a black-box model, and for real surgical applications, it is essential to understand the AI model and the reasoning behind its predictions. Therefore, we applied SHAP to make this AI model explainable.

**Supplementary table 2**. Performance obtained by the best configuration of each model in the 10-fold cross-validation to predict the outcome of the surgery. We trained Random Forest in the whole training set without scaling the data, and it obtained an RMSE of 0.2568, an MSE of 0.0660, and an MAE of 0.1148 in the test set. The performance achieved in the test set is right, allowing the prediction of the result of the surgery in real scenarios. Random Forest is also a black-box model, so we applied SHAP to make this AI model explainable.

| Model | Database  scaled | RMSE | MSE | MAE |
| --- | --- | --- | --- | --- |
| Random Forest | False | 0.1919 | 0.0382 | 0.0998 |
| Random Forest | True | 0.1919 | 0.0382 | 0.0999 |
| MLP | True | 0.1972 | 0.0403 | 0.1019 |
| MLP | False | 0.1969 | 0.0401 | 0.1026 |
| SVR | False | 0.1857 | 0.0355 | 0.1106 |
| Adaboost | True | 0.1843 | 0.0352 | 0.1151 |
| Adaboost | False | 0.1841 | 0.0351 | 0.1152 |
| SVR | True | 0.183 | 0.0346 | 0.1183 |
| KNN | False | 0.1819 | 0.0344 | 0.1216 |
| KNN | True | 0.1823 | 0.0345 | 0.123 |
| ElasticNet | True | 0.1829 | 0.0346 | 0.1239 |
| ElasticNet | False | 0.1819 | 0.0342 | 0.1241 |
| Tree | False | 0.2085 | 0.0448 | 0.1246 |
| Tree | True | 0.2085 | 0.0448 | 0.1246 |
| Linear regressor | False | 0.1862 | 0.0358 | 0.1263 |
| Linear regressor | True | 0.1862 | 0.0358 | 0.1263 |

**Supplementary table 3.** Results of Chi-square test for the numerical variables depending on the conversion to open surgery.

| Variable | Value | P-value |
| --- | --- | --- |
| Tumor location | 8.1308 | 0.0434 |
| Proximity to major vessel | 2.6702 | 0.1022 |
| HALS/Hybrid | 0.662 | 0.4159 |
| Readmission | 0.5866 | 0.4437 |
| Liver disease | 2.4298 | 0.4881 |
| Child Pugh type b | 0.2701 | 0.6033 |
| Gender | 0.1528 | 0.6959 |
| Resection | 0.4968 | 0.7801 |
| FCM surgery chemotherapy | 0.1348 | 0.9348 |
| Previous hepatectomy | 0 | 1 |

**Supplementary figure 1.**


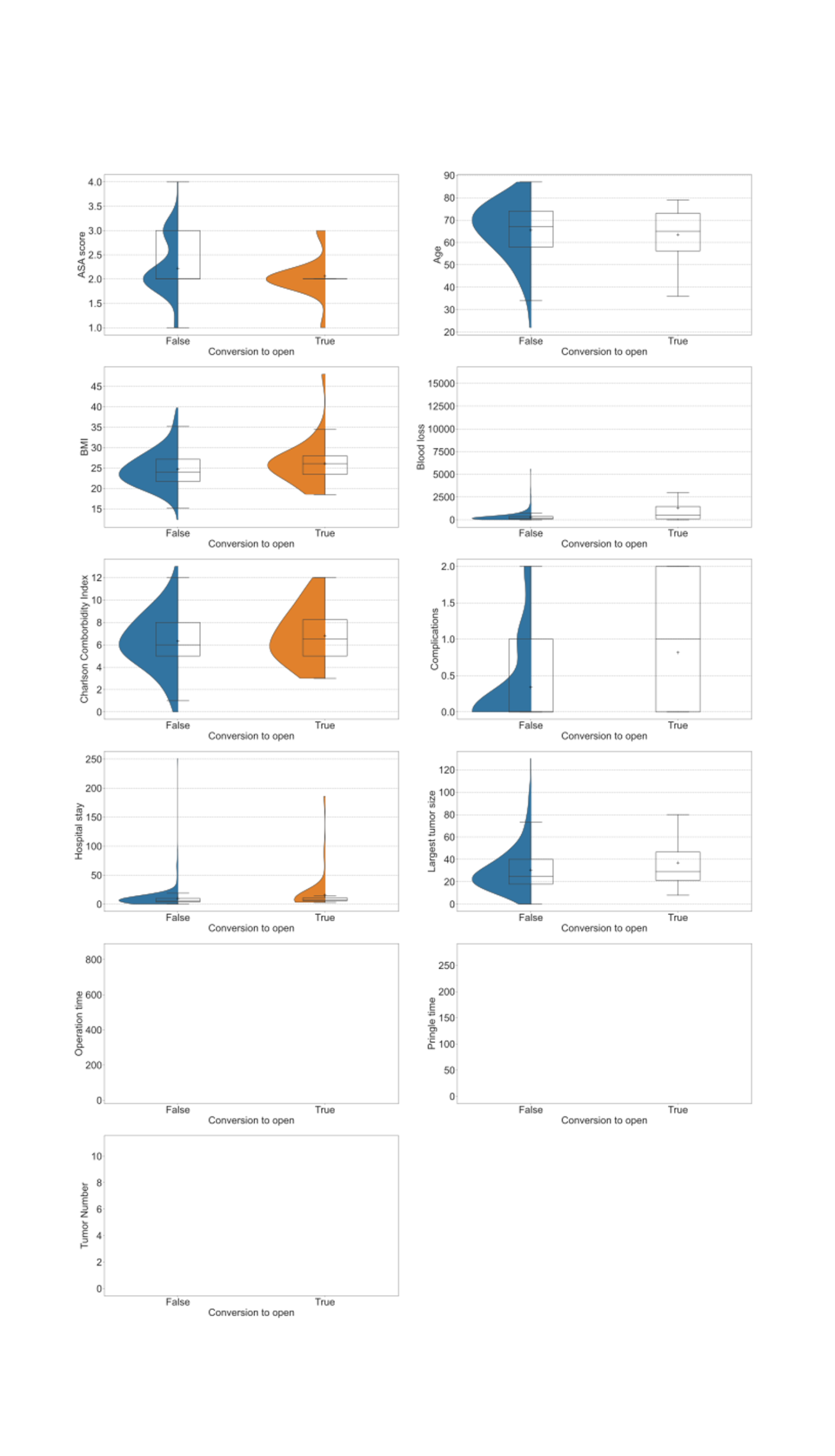

Supplement: Supplementary file 1 — Supplementary file1 (DOCX 586 KB) [file 464_2024_10681_MOESM1_ESM.docx]
